# Variability in Dietary Quality of Elementary School Lunch Menus with Changes in National School Lunch Program Nutrition Standards

**DOI:** 10.1093/cdn/nzaa138

**Published:** 2020-08-21

**Authors:** Kajal J Patel, Katie M Strait, Deana A Hildebrand, Lauren L Amaya, Jillian M Joyce

**Affiliations:** Department of Nutritional Sciences, Oklahoma State University, Stillwater, OK, USA; Department of Nutritional Sciences, Oklahoma State University, Stillwater, OK, USA; Department of Nutritional Sciences, Oklahoma State University, Stillwater, OK, USA; Department of Nutritional Sciences, Oklahoma State University, Stillwater, OK, USA; Department of Nutritional Sciences, Oklahoma State University, Stillwater, OK, USA

**Keywords:** National School Lunch Program, nutrition policy, dietary quality, child nutrition, adolescent nutrition, Healthy, Hunger-Free Kids Act, Child Nutrition Program, Child Nutrition Program Flexibilities

## Abstract

National School Lunch Program (NSLP) standards recently changed significantly. The Healthy, Hunger-Free Kids Act (HHFKA) presumably improved the dietary quality (DQ) of meals, whereas Child Nutrition Program (CNP) Flexibilities appear to decrease DQ. This variability has not been quantified. Our objective was to determine differences in DQ between elementary school lunch menus meeting NSLP standards: School Meal Initiative (SMI), HHFKA, CNP Flexibilities, and evidence-based best practices (BP). A base menu was portioned per NSLP standards and analyzed for nutrient content and DQ. Statistical analyses included 1-factor ANOVA, Kruskal–Wallis, and Dunnett's test. The BP menu had higher whole fruit and whole grain Healthy Eating Index scores than SMI (*P*s < 0.0083). The BP and HHFKA menus had higher refined grain and added sugars scores than SMI (*P*s < 0.0083). The SMI menu had lower total vegetable and saturated fat scores than all menus (*P*s < 0.0083). This study informs policy toward improving standards, positively affecting child health and academic performance through higher-DQ lunches.

## Introduction

Considering that adult and childhood obesity and type 2 diabetes rates have continued to rise for years relatively unimpeded (1, 2), while dietary quality (DQ) has remained relatively poor (3), a big-impact solution is needed. With diet being a major contributor to these disease conditions (4), focusing on nutrition is logical. Owing to the fact that 30.4 million US schoolchildren, over half of the US child population, participate in school nutrition programs each week (5, 6), continuing to improve child nutrition and the quality of their diets through school nutrition programs, like the National School Lunch Program (NSLP), could have a large impact on child and eventually adult health, obesity in particular.

The NSLP was established in 1946 with the goal of providing US schoolchildren with balanced and nutritious meals in order to combat malnutrition. The goal has since been modified to also include combatting obesity (7). The NSLP has evolved since its start in 1946, with some of the most recent nutrition standards including the School Meal Initiative (SMI, 1995; 7 CFR Part 210, 7 CFR Part 220), the Healthy, Hunger-Free Kids Act (HHFKA, 2012; 7 CFR Part 210), and the Child Nutrition Program Flexibilities (CNP Flexibilities, 2017; 7 CFR Part 210, 7 CFR Part 215, 7 CFR Part 220, 7 CFR Part 226).

The introduction of the HHFKA, in 2012, resulted in substantial changes to the SMI and other previous NSLP standards. These changes required schools to provide more whole grains, fruits, vegetables, lean protein, and low-fat dairy, while serving less fat, sugar, and sodium (8). These changes appear to improve the healthfulness of school meals. DQ assesses the healthfulness of a diet or eating pattern by comparing food and/or nutrient consumption to established recommendations for a healthy diet (4, 9). With the HHFKA in place, the DQ score, using the Healthy Eating Index (HEI) 2010, of a school lunch was reported to be between a 77 and an 82 out of 100, which was a 41% increase from previous standards (10, 11). Considering over half of US children participate in the NSLP and that the average US child's diet has a HEI score of 53 out of 100, which needs improvement according to the USDA Center for Nutrition Policy and Promotion (CNPP), the 41% increase in DQ of school lunches could be greatly benefitting a large proportion of US children (4, 11).

The CNP Flexibilities, introduced in 2017, are the most recent changes to the NSLP nutrition standards. These flexibilities allow schools to decrease whole grain provision, to provide higher sodium content, and to serve low-fat flavored milk options rather than fat free, as outlined in the HHFKA (12, 13). These changes could affect 3 major HEI scoring components through the offering of less whole grains, more saturated fat, and more sodium, lowering the overall DQ of school lunches. These changes could also negatively affect children, because research shows that increased dietary sodium, refined grain, and fat intakes among children lead to major health consequences (14–18).

Considering the average US child's DQ score is poor, further improvements in school meal DQ would greatly benefit children's overall DQ (4). In addition, improving DQ is important to focus on during childhood, because a higher DQ has been associated with healthier weight status, lower risk of chronic disease, and improved academic performance (9, 19–21). Recent changes to the nutrition standards via flexibilities appear to reverse some of the HHFKA increase in school lunch DQ; however, this reversal is yet to be verified. Thus, the purpose of this study was to determine the differences in nutrient content and DQ of elementary school lunch menus meeting NSLP nutrition standards including the SMI, HHFKA, and CNP Flexibilities, as well as one with school lunch evidence-based best practices (BP) implemented.

## Methods

### Study design and sampling method

This study used a cross-sectional content analysis to determine differences among 4 experimental menus created with the application of the 3 aforementioned different NSLP nutrition standards and of best practices: SMI, HHFKA, CNP Flexibilities, and BP. To establish a base menu, researchers conducted a search in September, October, and November of 2018 of school lunch menus in a southwestern state for one deemed typical, based on the expert opinion of 1 of the authors (JMJ). Researchers applied each of the 3 NSLP standards to the same base menu to create 3 of the 4 experimental menus. The fourth menu was created by applying BP, which were based on Dietary Guidelines for Americans (DGA) Healthy Meal Pattern Recommendations (22), Child and Adult Care Food Program Best Practices (23), and maximizing HEI 2015 scoring components (4).

The sample size included 30 school days (6 wk) for each experimental menu type based on a similar study by Joyce et al. (10). Power calculations were conducted to ensure that this sample size provided adequate power to detect significant differences between the experimental menus (Power and Sample Size Calculator, HyLown Consulting LLC, version 2018). Power was set at 0.80, and the level of significance was at 0.05, for a 2-tailed, 2-sample *t* test. Power analysis, based on the Joyce et al. (10) study, suggested the need for a sample size of 2 d. A sample size of 30 d was chosen to exceed that suggestion and cover most full-cycle menu lengths.

### NSLP nutrition standards

To create the 4 experimental menus, the 3 selected NSLP nutrition standards and BP were applied to the base menu by the graduate research assistant (KJP) and checked for content validity by registered dietitians and experts in school nutrition menus and programs (JMJ, DAH). **Table 1** provides a summary of the different nutrition standards. **Table 2** compares the 4 different NSLP standards, which shows the evolution across the past 3 standard systems. The information provided a guide to the researchers in creating the experimental menus and demonstrated how the menus differ. **Supplemental Tables 1**–**4** provide samples of the menus portioned per the 4 different NSLP standards.

**FIGURE 1:**
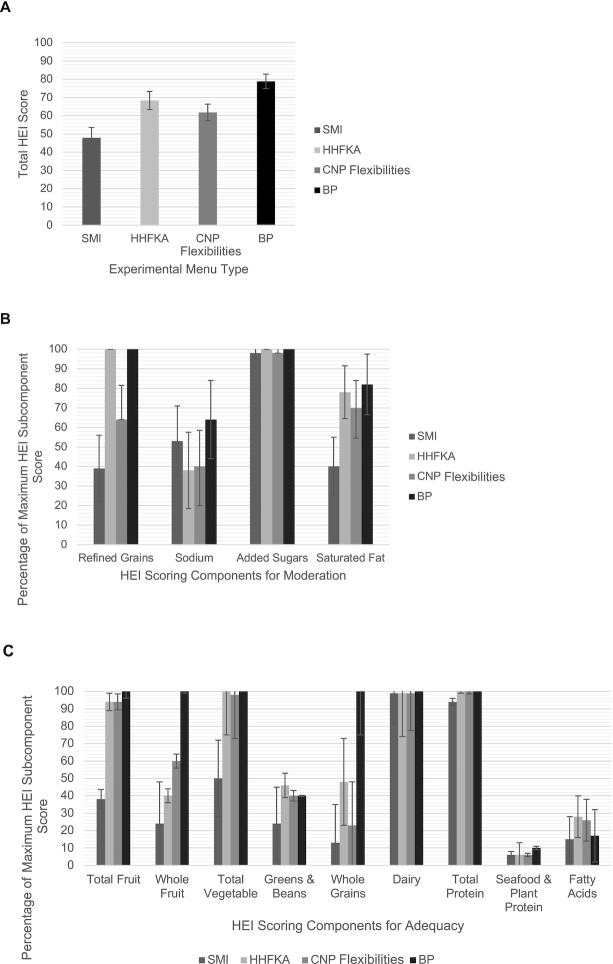
Comparison of total HEI scores and HEI subcomponent scores across experimental menu types. (A) HEI total score, (B) HEI scoring components for moderation, and (C) HEI scoring components for adequacy. Error bars represent standard deviation. BP, evidence-based best practices; CNP, Child Nutrition Program; HEI, Healthy Eating Index; HHFKA, Healthy, Hunger-Free Kids Act; SMI, School Meal Initiative.

**TABLE 1:** NSLP nutrition standards and best practices used to create the 4 experimental menus^1^

Component	SMI Traditional (Grades K–6, 4–12)^F^^,^^N^	HHFKA (Grades K–5)	CNP Flexibilities (Grades K–5)	Evidence-based best practices (Grades K–5)*
Fruit	½ cup combined per day,^F^ no subgroup specifications for vegetables	½ cup/d (2½ cups/wk)	No change	Increase provision, options with less added sugar
Vegetables		¾ cup/d (3¾ cups/wk); must have the following varieties throughout the week: dark green ½ cup, red/orange ¾ cup, beans/peas ½ cup (legumes), starchy ½ cup, other ½ cup	No change	Increase provision, choose options with less added sodium, do not add salt, no prefried, limit added fat and only use fats high in MUFA and PUFA
Meat/meat alternative	2 oz eq. min. per day^F^	1 oz eq. min. per day (8–10 oz/wk)	No change	Limit use of processed meats, no prefried, do not add salt, use leaner animal proteins (poultry, fish, eggs, low-fat dairy), limit red meats, increase use of plant-based proteins, limit added fat and only use fats high in MUFA and PUFA
Grains	1 serving/d^F^ (8/wk min.), whole grains encouraged	1 oz eq. min. per day (8–9 oz/wk), all grains must be whole grain rich	1 oz eq. min. per day (8–9 oz/wk), half of grains must be whole grain rich	Use 100% whole grains, limit refined grains, use low-sodium chips/crackers, limit added fat and only use fats high in MUFA and PUFA, no prefried, do not include grain-based desserts
Milk	1 cup^F^ (variety of fat contents allowed, flavor not restricted)	1 cup (fat-free or 1% low-fat plain, fat-free flavored)	1 cup fat-free or low-fat plain or flavored	Use only unflavored low-fat or fat-free dairy
Calories	664^N^	Average for a 5-d week must be between 550 and 650	No change	No standard provided
Sodium	No standard provided	Target 1: ≤1230 mgTarget 2: ≤935 mgFinal target: ≤640 mg	Stopped at target 1: ≤1230 mg; no further reductions	Decrease content
Total fat	22 g^N^	No standard provided	No change	Decrease content
Saturated fat	7 g^N^	<10% of calories	No change	Decrease content
*Trans* fat	No standard provided	No *trans* fat permitted	No change	No standard provided
Vitamin A	224 RE^N^	No standard provided	No change	No standard provided
Vitamin C	15 mg^N^	No standard provided	No change	No standard provided
Iron	3.5 mg^N^	No standard provided	No change	No standard provided
Calcium	286 mg^N^	No standard provided	No change	No standard provided

^1^CNP, Child Nutrition Program; HHFKA, Healthy, Hunger-Free Kids Act; RE, retinol equivalents; SMI, School Meal Initiative; 1 oz = 28 g; 1 cup = 237 mL.

F Food Based Menu Planning Approach for SMI.

N Nutrient Based Menu Planning Approach for SMI.

* In addition to HHFKA standards.

**TABLE 2:** Changes in NSLP nutrition standards across versions^1^

	SMI	HHFKA (reference)	CNP Flexibilities	Evidence-based best practices
Year policy implemented in schools or best practices established	1995	2012	2017	DGA 2015, CACFP best practices 2016, unpublished review 2017
Fruits	3¾ cup less per week, does not have to offer fruit and vegetables separately, no vegetable subgroups	½ cup/d (2½ cups/wk)	Remains the same	Increase provision, options with less added sugar
Vegetables	As above	¾ cup/d (3¾ cups/wk), must have varieties throughout the week: dark green ½ cup, red/orange ¾ cup, beans/peas ½ cup (legumes), starchy ½ cup, other ½ cup	Remains the same	Increase provision, choose options with less added sodium, do not add salt, no prefried, limit added fat and only use fats high in MUFA and PUFA
Meat/meat alternative	Added weekly maximum	1 oz eq. min. per day (8–10 oz/wk)	Remains the same	Limit use of processed meats, no prefried, do not add salt, use leaner animal proteins (poultry, fish, eggs, low-fat dairy), limit red meats, increase use of plant-based proteins, limit added fat and only use fats high in MUFA and PUFA
Grains	Minimum amount remained the same, added weekly maximum, only encouraged whole grains	1 oz eq. min. per day (8–9 oz/wk), all grains must be whole grain rich	Decrease whole grain provision by half	Use 100% whole grains, limit refined grains, use low-sodium chips/crackers, limit added fat and only use fats high in MUFA and PUFA, no prefried, do not include grain-based desserts
Milk	No change in amount, flavor and fat not restricted	1 cup (fat-free or 1% low-fat plain, fat-free flavored)	Allows low fat and flavored	Use only unflavored low-fat or fat-free dairy
Calories	No range, 14 calories above the HHFKA upper range	Average for a 5-d week must be between 550 and 650	Remains the same	No standard provided
Sodium	No standard	Target 1: ≤1230 mgTarget 2: ≤935 mgFinal target: ≤640 mg	Target timeline extended, final target eliminated	Decrease
Total fat	Provided standard that was eliminated	No standard provided	Remains the same	Decrease
Saturated fat	9 g = 9.8% of calories, slightly below the HHFKA limit	<10% of calories	Remains the same	Decrease
*Trans* fat	No standard	No standard provided	Remains the same	No standard provided
Vitamin A	Standard covered in HHFKA vegetable variety	No standard provided	Remains the same	No standard provided
Vitamin C	Standard covered in HHFKA vegetable variety	No standard provided	Remains the same	No standard provided
Iron	Standard met by food group requirements for all NSLP versions	No standard provided	Remains the same	No standard provided
Calcium	Standard met by food group requirements for all NSLP versions	No standard provided	Remains the same	No standard provided

1 CACFP, Child and Adult Care Food Program; CNP, Child Nutrition Program; DGA, Dietary Guidelines for Americans; HHFKA, Healthy, Hunger-Free Kids Act; NSLP, National School Lunch Program; SMI, School Meal Initiative; 1 oz = 28 g; 1 cup = 237 mL.

### DQ

Once the standards had been applied to the experimental menus, the portioned experimental menus were entered into ESHA Food Processor Nutrient Analysis Software (version 10.11.0, 2017) to determine nutrient content. DQ was then determined using the HEI 2015 (USDA CNPP) (4). The HEI scoring method is commonly used to assess DQ in the United States, appropriate for this population, and considered a valid and reliable measure of DQ based on the 2005 and 2010 versions (24–27). The total score ranges from 0 to 100 points. A higher HEI score indicates higher DQ. Scoring subcomponents of the HEI include fruits, vegetables, whole grains, greens and beans, dairy, total protein, refined grains, sodium, added sugars, and saturated fats. Scores for subcomponents range from 5 to 10 points. The HEI also evaluates diets for balance, variety, adequacy, and moderation, along with food groups (4, 25).

HEI scores were calculated using a Microsoft Excel calculator created by 1 of the authors (JMJ). Formulas in the calculator first standardized the raw food and nutrient content provided by the lunch to the equivalent content per 1000 calories using proportions. Next, the formulas determined what percentage of the amount of a food or nutrient needed to obtain a maximum score was provided by the lunch's 1000-calorie equivalent. Once the percentage of the amount of a food or nutrient needed to obtain a maximum score was determined, this same percentage was taken of the maximum point score for that HEI subcomponent (i.e., 5 or 10 points) to determine the score the lunch received for that food or nutrient HEI scoring subcomponent. Finally, researchers reviewed all total and subcomponent HEI scores calculated to ensure they did not exceed the maximum possible score for that HEI scoring component. If the score exceeded the maximum possible, the score was adjusted to be the maximum score.

### Statistical analysis

Descriptive statistics used included means ± SDs. A 1-factor ANOVA was used to determine if differences existed in nutrient content and DQ between the 4 different experimental menus. Dunnett's test was performed for pairwise comparisons. With correction for multiple comparisons, the level of significance was set at *P* < 0.0083. Assumptions were checked using a Kolmogorov–Smirnov test for normality and Brown–Forsythe and Levene's tests for equality of variance. Variables found to be nonnormal were transformed using log and inverse transformations. If variables continued to be nonnormal, a Kruskal–Wallis test was performed to determine significant differences between experimental menus. Effect size to determine clinical significance was determined using eta squared.

## Results

### Content of nutrients required for monitoring by the NSLP

**Table 3** shows descriptive statistics for and significant differences in nutrients required for monitoring by the NSLP. Menus significantly differed in calories (η^2^ = 0.121), saturated fat (η^2^ = 0.271), *trans* fat (η^2^ = 0.186), and sodium (η^2^ = 0.145) content. The BP menu was 16% lower in calories than the HHFKA menu (mean difference = 99 calories, *P* < 0.0083) and 15% lower than the CNP Flexibilities menu (mean difference = 96 calories, *P* < 0.0083). For saturated fat, the SMI menu was 40% and 46% higher than the HHFKA and BP menus, respectively (mean differences = 2.5 g and 4.0 g, respectively; *P*s < 0.0083). The BP menu was 46% lower in saturated fat than the SMI menu (mean difference = 4.0 g, *P* < 0.0083) and 35% lower than the CNP Flexibilities menu (mean difference = 2.2 g, *P* < 0.0083). For *trans* fat, the BP menu was 75% lower than the SMI menu and 67% lower than the HHFKA and CNP Flexibilities menus (mean differences = 0.3 g, 0.2 g, and 0.2 g, respectively; *P*s < 0.0083). In relation to sodium, the BP menu was 34% lower than the HHFKA menu (mean difference = 385 mg, *P* < 0.0083) and 32% lower than the CNP Flexibilities menu (mean difference = 353 mg, *P* < 0.0083).

**TABLE 3:** Comparison of nutrient content between experimental menus^1^

Nutrient	SMI	HHFKA	CNP Flexibilities	BP
Calories*	601 ± 134^a,b^	628 ± 111^b^	625 ± 110^b^	529 ± 72^a^
Protein, g	30.2 ± 12.4^a^	30.7 ± 4.7^a,b^	30.6 ± 4.4^a,b^	34.7 ± 5.0^b^
Carbohydrate, g	71.3 ± 19.5	87.3 ± 20.0	82.9 ± 17.5	73.9 ± 11.3
Total fiber, g	5.2 ± 2.4^a^	8.0 ± 3.2^b^	7.6 ± 3.1^a,b^	9.6 ± 3.5^b^
Sugar, g	28.3 ± 9.9^a^	39.9 ± 8.3^b^	38.7 ± 8.4^b^	27.5 ± 4.2^a^
Added sugar, g	5.3 ± 6.3	5.2 ± 6.0	5.7 ± 6.6	0.4 ± 1.0
Total fat, g	21.9 ± 7.5^a^	18.3 ± 6.8^a^	20.0 ± 7.4^a^	11.9 ± 4.7^b^
SFAs,* g	8.7 ± 2.7^a^	6.2 ± 2.2^b,c^	6.9 ± 2.5^a,b^	4.7 ± 2.2^c^
MUFAs, g	6.0 ± 2.9^a^	5.0 ± 2.4^a,b^	5.6 ± 2.5^a^	3.3 ± 1.5^b^
PUFAs, g	3.0 ± 2.6	3.2 ± 2.6	3.4 ± 2.8	2.1 ± 1.2
*Trans* fat,* g	0.4 ± 0.3^a^	0.3 ± 0.2^a^	0.3 ± 0.2^a^	0.1 ± 0.4^b^
Cholesterol, mg	76.4 ± 68.9	55.6 ± 15.7	60.5 ± 15.1	57.2 ± 16.5
Vitamin A, IU	1016 ± 1163	3167 ± 4445	3197 ± 4524	3442 ± 5403
Vitamin B-6, IU	0.49 ± 0.32	0.56 ± 0.25	0.75 ± 0.34	0.60 ± 0.31
Vitamin B-12, μg	2.01 ± 0.70	1.92 ± 0.69	1.87 ± 0.75	1.87 ± 0.57
Vitamin C, mg	8.94 ± 10.93	19.06 ± 16.01	18.64 ± 16.39	19.95 ± 18.87
Vitamin D, IU	59.9 ± 63.5	54.1 ± 57.9	55.9 ± 59.9	45.1 ± 56.3
Vitamin E, mg	1.46 ± 1.12	1.74 ± 1.24	1.71 ± 1.24	1.60 ± 1.00
Folate, μg	73.0 ± 44.0	76.3 ± 42.5	80.1 ± 42.4	109.8 ± 57.3
Vitamin K, μg	16.3 ± 20.9	26.9 ± 26.9	27.3 ± 27.7	27.6 ± 27.9
Calcium, mg	477 ± 156	525 ± 135	524 ± 147	536 ± 166
Iron, mg	3.48 ± 1.17	3.78 ± 1.15	3.75 ± 1.67	3.32 ± 1.13
Magnesium, mg	82.9 ± 26.3	104.7 ± 26.5	98.4 ± 28.3	119.2 ± 31.5
Phosphorus, mg	525 ± 152	567 ± 118	553 ± 127	614 ± 117
Potassium,* mg	893 ± 191^a^	1179 ± 246^b^	1142 ± 235^b^	1133 ± 176^b^
Sodium,* mg	943 ± 370^a,b^	1135 ± 415^b^	1103 ± 388^b^	750 ± 332^a^
Zinc, mg	3.83 ± 1.83	4.31 ± 2.09	4.27 ± 2.04	3.77 ± 1.22

^1^Values are means ± SDs. Means in a row without a common superscript letter are significantly different. *Nutrients monitored by the National School Lunch Program. BP, evidence-based best practices; CNP, Child Nutrition Program; HHFKA, Healthy, Hunger-Free Kids Act; SMI, School Meal Initiative.

### Content of other macro- and micronutrients of concern

Table 3 also shows descriptive statistics for and significant differences in additional nutrients monitored indirectly by the NSLP. Menus significantly differed in protein (η^2^ = 0.156), total fiber (η^2^ = 0.217), sugar (η^2^ = 0.327), total fat (η^2^ = 0.247), MUFAs (η^2^ = 0.163), and potassium (η^2^ = 0.226). Protein content in the BP menu was 15% higher than in the SMI menu (mean difference = 4.5 g, *P* < 0.0083). For fiber, the SMI menu was 35% lower than the HHFKA menu (mean difference = 2.8 g, *P* < 0.0083) and 46% lower than the BP menu (mean difference = 4.4 g, *P* < 0.0083). For sugar content, the SMI menu was 29% lower than the HHFKA menu (mean difference = 11.6 g, *P* < 0.0083) and 27% lower than the CNP Flexibilities menu (mean difference = 10.4 g, *P* < 0.0083). The BP menu was 31% lower in sugar than the HHFKA menu (mean difference = 12.4 g, *P* < 0.0083) and 29% lower than the CNP Flexibilities menu (mean difference = 11.2 g, *P* < 0.0083). The total fat content for the BP menu was 46% lower than the SMI menu (mean difference = 10.0 g, *P* < 0.0083), 35% lower than the HHFKA menu (mean difference = 6.4 g, *P* < 0.0083), and 41% lower than the CNP Flexibilities menu (mean difference = 8.1 g, *P* < 0.0083). MUFA content for the BP menu was 45% lower than for the SMI menu (mean difference = 2.7 g, *P* < 0.0083) and 41% lower than for the CNP Flexibilities menu (mean difference = 2.3 g, *P* < 0.0083). For potassium, the SMI menu was 24% lower than the HHFKA menu (mean difference = 286.4 mg, *P* < 0.0083), 22% lower than the CNP Flexibilities menu (mean difference = 249.4 mg, *P* < 0.0083), and 21% lower than the BP menu (mean difference = 236.4 mg, *P* < 0.0083).

### DQ

**Table 4** and **Figure 1** show the comparison of DQ, as HEI 2015 scores and HEI subcomponents, between the experimental menus. Menus significantly differed in total HEI score (η^2^ = 0.582) and subcomponent scores including total fruit (η^2^ = 0.121), whole fruit (η^2^ = 0.332), total vegetable (η^2^ = 0.344), whole grains (η^2^ = 0.456), refined grains (η^2^ = 0.535), added sugars (η^2^ = 0.071), and saturated fat (η^2^ = 0.243). The total HEI score for the SMI menu was 30% lower than for the HHFKA menu (mean difference = 20.5, *P* < 0.0083), 22% lower than for the CNP Flexibilities menu (mean difference = 13.9, *P* < 0.0083), and 39% lower than for the BP menu (mean difference = 31.0, *P* < 0.0083). For subcomponent scores, the total fruit score of the BP menu was 6% higher than that of the HHFKA menu (mean difference = 0.3, *P* < 0.0083). Whole fruit in the BP menu was 317% higher than in the SMI menu (mean difference = 3.8, *P* < 0.0083), 150% higher than in the HHFKA menu (mean difference = 3.0, *P* < 0.0083), and 66% higher than in the CNP Flexibilities menu (mean difference = 2.0, *P* < 0.0083). For total vegetable, the SMI menu was 50% lower than the HHFKA menu (mean difference = 2.5, *P* < 0.0083), 49% lower than the CNP Flexibilities menu (mean difference = 2.4, *P* < 0.0083), and 50% lower than the BP menu (mean difference = 2.5, *P* < 0.0083). The whole grains score for the BP menu was 669% higher than for the SMI menu (mean difference = 8.7, *P* < 0.0083), 108% higher than for the HHFKA menu (mean difference = 5.2, *P* < 0.0083), and 335% higher than for the CNP Flexibilities menu (mean difference = 7.7, *P* < 0.0083). For the refined grains score, the BP menu was 56% higher than the CNP Flexibilities menu (mean difference = 3.6, *P* < 0.0083) and 156% higher than the SMI menu (mean difference = 6.1, *P* < 0.0083), whereas no difference existed between the BP and HHFKA menus. For the added sugars subcomponent score, the BP menu was 2% higher than the CNP Flexibilities menu and the SMI menu (mean differences = 0.2, *P*s < 0.0083), whereas no difference existed between the BP and HHFKA menus. The HHFKA menu was also 2% higher for the added sugars score than the SMI menu and the CNP Flexibilities menu (mean differences = 0.2, *P*s < 0.0083). For the final HEI subcomponent score of saturated fat, the SMI menu was 49% lower than the HHFKA menu (mean difference = 3.8, *P* < 0.0083), 43% lower than the CNP Flexibilities menu (mean difference = 3.0, *P* < 0.0083), and 51% lower than the BP menu (mean difference = 4.2, *P* < 0.0083).

**TABLE 4:** Comparison of dietary quality between experimental menus as HEI 2015 total scores and subcomponents^1^

HEI component scores	SMI	HHFKA	CNP Flexibilities	BP
Total HEI score	47.9 ± 11.3^a^	68.4 ± 10.0^b^	61.8 ± 9.2^b^	78.9 ± 7.9^c^
Total fruit	1.9 ± 2.4^a^	4.7 ± 0.4^a,b^	4.7 ± 0.4^a,b^	5.0 ± 0.1^b^
Whole fruit	1.2 ± 2.2^a^	2.0 ± 2.5^a^	3.0 ± 2.5^a^	5.0 ± 0.0^b^
Total vegetable	2.5 ± 2.1^a^	5.0 ± 0.7^b^	4.9 ± 0.3^b^	5.0 ± 0.03^b^
Dark greens/legumes	1.2 ± 2.2	2.3 ± 2.5	2.0 ± 2.5	2.0 ± 2.5
Whole grains	1.3 ± 3.5^a^	4.8 ± 5.0^a^	2.3 ± 4.3^a^	10.0 ± 0.0^b^
Dairy	9.9 ± 0.4	9.9 ± 0.2	9.9 ± 0.3	10.0 ± 0.0
Total protein	4.7 ± 0.2	5.0 ± 0.7	5.0 ± 0.1	5.0 ± 0.1
Seafood/plant protein	0.3 ± 1.3	0.3 ± 1.2	0.3 ± 1.2	0.5 ± 1.5
Fatty acid ratio	1.5 ± 2.8	2.8 ± 4.0	2.6 ± 3.7	1.7 ± 2.5
Refined grains	3.9 ± 3.4^a^	10.0 ± 0.0^b^	6.4 ± 3.5^a^	10.0 ± 0.0^b^
Sodium	5.3 ± 3.6	3.8 ± 3.9	4.0 ± 3.7	6.4 ± 4.0
Added sugars	9.8 ± 0.6^a^	10.0 ± 0.0^b^	9.8 ± 0.6^a^	10.0 ± 0.0^b^
Saturated fat	4.0 ± 3.0^a^	7.8 ± 2.7^b^	7.0 ± 2.8^b^	8.2 ± 3.1^b^

^1^Values are means ± SDs. Means in a row without a common superscript letter are significantly different. BP, evidence-based best practices; CNP, Child Nutrition Program; HEI, Healthy Eating Index; HHFKA, Healthy, Hunger-Free Kids Act; SMI, School Meal Initiative.

## Discussion

The purpose of this cross-sectional content analysis was to investigate the differences in DQ of school lunch menus that meet the various recent NSLP nutrition standards. Applying best practices and HHFKA nutrition standards both resulted in higher HEI subcomponent scores for refined grains and added sugars than the SMI. The SMI menu had the lowest HEI score for total vegetable and saturated fats compared with the HHFKA, CNP Flexibilities, and BP menus. Thus, policy changes over time have significantly affected DQ of school lunches, related to refined grain, added sugars, total vegetable, and saturated fat HEI subcomponents.

High DQ, as evidenced by a high HEI score, is important in childhood. The HEI assesses DQ by determining how well a person's diet aligns with the DGA (9, 23–25). Measuring DQ is a more true-to-life approach to assess healthfulness of a diet and nutrition provided because it takes into consideration the whole diet, as compared with focusing on individual nutrients. It is less practical to look at individual nutrients because people do not, for the most part, consume nutrients individually. The HEI is one of the most commonly used measures of DQ, because it is appropriate for anyone to whom the DGA apply in the United States of 2 y and older (9, 23–25). DQ is important to assess during childhood, because a lower HEI score, and thus lower DQ, is associated with higher risk of overweight, obesity, mortality, and chronic disease in childhood and on into adulthood (9, 20, 28). A higher HEI score is also associated with improved academic performance (21). Federal CNPs, including the NSLP if used by children, could be contributing significantly to their daily nutrition and HEI score. Thus, knowing how the changes in NSLP policy affect school lunch DQ is of great importance.

This is the first study to our knowledge to investigate the impact on school lunch DQ of multiple recent changes in NSLP nutrition policy. According to a study by the USDA Food and Nutrition Service, HEI scores of school lunches increased significantly between school years 2009 and 2010 and again between 2014 and 2015. The HEI score for NSLP-qualifying school lunches increased from 57.9 to 81.5 out of 100 (11). The current study adds to the idea that NSLP policy changes moved in a positive direction with implementation of the HHFKA. Another study by Joyce et al. (10) examined differences between a typical school lunch menu, meeting baseline HHFKA NSLP nutrition standards, and a best practice school lunch menu, optimizing nutrition. This study found that applying best practices to a school lunch menu could significantly further improve the HEI score of NSLP-qualifying school lunches (11). The current study results are consistent with and add to those of the Joyce et al. (10) study in that the HHFKA policy changes improved the DQ of school lunches, but there is additional room for further improvement.

With policy changes improving the DQ of school lunches, numerous perceived barriers and challenges did and still do exist. Significant areas of concern include decreases in NSLP participation rates and increases in food waste from serving healthier food items with lower child acceptability (29). Studies conducted by Vaudrin et al. (30) and the USDA (11) reported participation rates did not decrease, but steadily increased after implementation of the HHFKA. In addition, Schwartz et al. (31) measured plate waste from 12 urban schools with lunches meeting NSLP nutrition standards, from 2012 (before the HHFKA) to 2014 (after HHFKA implementation), and found that the selection and consumption of fruits and entrée choices significantly increased. Fruit selection increased from 54% to 66%, whereas fruit consumption remained at 74%. Entrée consumption significantly increased from 70% to 84%. In addition, vegetable consumption significantly increased from 46% to 64%. Other studies show similar findings with increased selection and consumption of healthier meals (32–35). These findings indicate that increased DQ of meals, under the HHFKA, was not associated with decreased participation rates and increased plate waste. On the contrary, most studies show improved participation rates, increased selection, and increased consumption of higher-DQ school lunches after 2014 and HHFKA implementation. Thus, changes in school nutrition guidelines made to favor higher DQ may positively affect school nutrition program performance, in addition to the health and academic performance of schoolchildren.

### Strengths

Strengths of this study include that the NSLP nutrition standards were only applied to 1 base menu, as opposed to 4 different base menus. This single base menu ensures that differences in DQ are not due to different menus and the differences inherently in those menus. All experimental menus were created for the same season to eliminate seasonal variations. For example, best practices encourage fresh fruit and vegetable consumption, which could include seasonal items to lower cost and improve food quality. Furthermore, the base menu used for this study was a true-to-life menu, not research-created, which helps eliminate bias and improve practicality. In addition, DQ was determined using the HEI 2015, which has been shown to be valid and reliable (20). Another strength was that researchers were transparent and used the same portioning and nutrient analysis assumptions for each menu, favoring higher DQ for all menus. Furthermore, only 2 researchers entered experimental menus for analysis, and 1 additional researcher reviewed all analyses to help ensure consistency and reduce intrarater variability. Lastly, power calculations ensured the sample size was adequate to detect significant differences.

### Limitations

A limitation of this study includes the cross-sectional design, which is considered a weaker observational study design; however, this design best met the purpose of this study. Another limitation includes possible misinterpretation of the NSLP nutrition policy standards. However, the interpretation by researchers was made transparently and consistently throughout the methodology. A limitation within the nutrient analysis of experimental menus includes the use of ESHA Food Processor, which does not have CNP labeled and approved versions of food items. However, where possible, USDA standard references were used to represent food items on the menu and were consistently used across all experimental menus to represent the same food items. In addition, consistent food codes were used for similar food items, and all researchers applied consistent assumptions. Furthermore, this study used a theoretical design and theoretical menus, which were not perfectly true to life. However, the use of 4 different actual menus would have resulted in the comparison of different base menus, and thus differences seen between standards might have been due to the base menus and not the standards themselves. Finally, the base menu came from a single southwestern state, and despite being deemed typical by a registered dietitian and expert in school lunch menu analysis, it may not be representative of typical menus in other regions of the United States.

### Importance of findings

The results of the current study can be used to inform NSLP policy. In 2012, the HHFKA led to significant and larger improvements in DQ of school lunches relative to the SMI, especially in regards to total fruit and vegetables. More recently, in 2017, the CNP Flexibilities did not significantly decrease DQ, but do appear to be trending toward decreased DQ from that of the HHFKA, because the flexibilities resulted in fewer improvements over previous versions than the HHFKA. Despite HHFKA improvements, further significant improvement in the DQ of NSLP-qualifying school lunches could be made, as evidenced by the BP menu having the highest DQ. Thus, future NSLP policy should seek to continue to improve nutrition standards and the resulting DQ of school lunches.

### Conclusions

The results of the current study showed that great improvements were made in the DQ of school lunches from HHFKA changes in NSLP policy, but there are possibly more meaningful improvements yet to be made. This study provides important information for guiding future policy toward further improving NSLP nutrition standards in their mission to provide healthy food to children, combatting malnutrition and obesity. Continuing to improve NSLP policy has the potential to affect the health, academic performance, and future of US children through higher-DQ school lunches.

## Supplementary Material

nzaa138_Supplemental_File

## Notes

Supported by Oklahoma State University Vice President for Research (to JMJ).

Author disclosures: the authors report no conflicts of interest.

Supplemental Tables 1–4 are available from the “Supplementary data” link in the online posting of the article and from the same link in the online table of contents at https://academic.oup.com/cdn/.

Abbreviations used: BP, evidence-based best practices; CNP, Child Nutrition Program; CNPP, Center for Nutrition Policy and Promotion; DGA, Dietary Guidelines for Americans; DQ, dietary quality; HEI, Healthy Eating Index; HHFKA, Healthy, Hunger-Free Kids Act; NSLP, National School Lunch Program; SMI, School Meal Initiative.
