# Supplementary material for: Variability in Dietary Quality of Elementary School Lunch Menus with Changes in National School Lunch Program Nutrition Standards
Source: Curr Dev Nutr. 2020 Aug 21;4(9):nzaa138. doi: 10.1093/cdn/nzaa138 (PMC7485789; doi:10.1093/cdn/nzaa138)
Supplement: nzaa138_Supplemental_File [file nzaa138_supplemental_file.docx]

Supplementary Table 1. Six Weeks of Portioned Lunch Menu for Grades K-5 Applying SMI Nutrition Standards

Week #: 1

| **Component** | **Mon (9/24/18)** | **Tues (9/25/18)** | **Wed (9/26/18)** | **Thurs (9/27/18)** | **Fri**  **(9/28/19)** |
| --- | --- | --- | --- | --- | --- |
| **Fruit &/or**  **Veg (1/2c)** | 1/2c French fries | 1/2c ranchero beans | 1 med. baked potato | 1/2c banana slices | 1c romaine lettuce, 1T LF Italian dressing |
| **Meat (2oz or 14oz protein)** | 2oz ground beef | 2oz ground beef, 2T cheese sauce | 14oz protein between chili and cheese (1/2 c. chili ¾ oz. cheese) | 14oz protein from nuggets | 14oz protein from cheese pizza |
| **Grain (2oz per day)** | 1 hamburger bun | 2oz WG nacho chips | 2oz chocolate chip cookie | 2oz 1 roll, 1t butter | 30g CHO from pizza |
| **Milk (1c)** | 1c milk 2% | 1c chocolate milk 2% | 1c milk 2% | 1c chocolate milk 2% | 1c milk 2% |

Week #: 2

| **Component** | **Mon (10/1/18)** | **Tues (10/2/18 )** | **Wed (10/3/18 )** | **Thurs (10/4/18 )** | **Fri**  **(10/5/18)** |
| --- | --- | --- | --- | --- | --- |
| **Fruit &/or**  **Veg (1/2c)** | 1/2c apple slices | 1/2c fresh celery 1 T LF ranch | 1c tomato soup | 1/2c banana slices | 1c romaine lettuce, 1T LF Caesar dressing |
| **Meat (2oz or 14oz protein)** | 2oz ground beef | 14g protein between chili and cheddar cheese (0.75 c. chili & 0.25 oz. cheese) | 2oz American cheese | 14g protein chicken tender | 14oz protein from Pepperoni pizza |
| **Grain (2oz per day)** | 1 hamburger bun | 2oz Fritos chips WG | 2oz bread | 2oz waffles and 1T syrup | 30g from pizza |
| **Milk (1c)** | 1c milk 2% | 1c chocolate milk 2% | 1c milk 2% | 1c chocolate milk 2% | 1c milk 2% |

Week #: 3

| **Component** | **Mon (10/8/18)** | **Tues (10/8/18)** | **Wed (9/19/18)** | **Thurs (10/11/18)** | **Fri (10/11/18)** |
| --- | --- | --- | --- | --- | --- |
| **Fruit &/or**  **Veg (1/2c)** | 1/2c potato wedges | 1/2c refried beans | 1/2c marinara sauce  1/2c canned pineapple | 1/2c banana slices | 1c iceberg lettuce, 1T Italian dressing |
| **Meat (2oz or 14oz protein)** | 2oz ground beef | 2oz ground beef (with 1/8 tsp taco seasoning) | 2oz ground beef | 14g protein chicken tenders | 2oz moz cheese, 2T pizza sauce |
| **Grain (2oz per day)** | 1 hamburger bun | 2oz tortillas | 1c spaghetti | 2oz garlic toast | 2oz flatbread |
| **Milk (1c)** | 1c milk 2% | 1c chocolate milk 2% | 1c milk 2% | 1c chocolate milk 2% | 1c milk 2% |

Week #: 4

| **Component** | **Mon (10/15/18 )** | **Tues (10/16/18 )** | **Wed (11/7/18 )** | **Thurs (11/8/18 )** | **Fri**  **(11/9/18 )** |
| --- | --- | --- | --- | --- | --- |
| **Fruit &/or**  **Veg (1/2c)** | 1/2c French fries | 1/2c canned peaches | 1/2c broccoli 1T LF ranch | 1/2c berries (1/4c. strawberries 1/4c blueberries) mixed frozen | 1/2c. sweet potato fries |
| **Meat (2oz or 14oz protein)** | 14g protein BBQ pork rib | 1.5oz chicken 2 oz. black beans | 14g protein tangerine chicken | 4oz vanilla yogurt, 1oz mozzarella cheese stick | 14g protein between refried beans and cheese |
| **Grain (2oz per day)** | 2oz hoagie bun | 2oz tortilla and 1/4c white rice | 1c cooked rice | 1/2c. Granola WG | 2oz tortilla and 1/4c white rice |
| **Milk (1c)** | 1c milk 2% | 1c chocolate milk 2% | 1c milk 2% | 1c chocolate milk 2% | 1c milk 2% |

Week #: 5

| **Component** | **Mon (11/12/18)** | **Tues (11/13/18)** | **Wed (11/14/18)** | **Thurs (11/15/18)** | **Fri (11/16/18)** |
| --- | --- | --- | --- | --- | --- |
| **Fruit &/or**  **Veg (1/2c)** | 1/2c apple slices | 1/2c canned peaches | 1c lettuce, 1T LF ranch | 1/2c bananas | 1/2c tropical fruit |
| **Meat (2oz or 14oz protein)** | 14g protein breaded chicken | 14g protein from cheese pizza | 2oz ground beef, 1/8c sloppy joe sauce | 2oz Swedish meatballs | 14oz protein from cheese |
| **Grain (2oz per day)** | 2oz hamburger bun | 30g CHO from pizza | 2oz hamburger bun | 1c cooked rice | 30g CHO macaroni pasta |
| **Milk (1c)** | 1c milk 2% | 1c chocolate milk 2% | 1c milk 2% | 1c chocolate milk 2% | 1c milk 2% |

Week #: 6

| **Component** | **Mon (10/8/19)** | **Tues (10/9/18)** | **Wed (10/10/18)** | **Thurs (10/11/18)** | **Fri (10/12/18)** |
| --- | --- | --- | --- | --- | --- |
| **Fruit &/or**  **Veg (1/2c)** | 1/2c potato wedges | 1/2c refried beans | 1/2c broccoli, 1T cheese sauce | 1/2c marinara sauce | 1/2c tropical fruit mix |
| **Meat (2oz or 14oz protein)** | 2oz grilled chicken | 1oz shredded chicken, 1oz cheddar cheese | 14oz protein grilled chicken leg | 2oz mozzarella cheese | 1.5oz shredded chicken, 0.5oz cheddar cheese, 2T BBQ sauce |
| **Grain (2oz per day)** | 2oz hamburger bun | 2oz tortillas | 1c cooked rice | 1c ziti pasta  1 piece garlic toast | 2oz nacho chips WG |
| **Milk (1c)** | 1c milk 2% | 1c chocolate milk 2% | 1c milk 2% | 1c chocolate milk 2% | 1c milk 2% |

**Portioning Notes:**

Fruits:

- 1T low-fat ranch with vegetables
- 1/2t butter with cooked vegetables
- Choices were based on which compliments entrée the best

Grains:

- Did not use whole grains on menu because whole grains are encouraged but not required under SMI nutrition standards.
  - Unavailable for schools, easier to do non whole grain products, perceptions that children will not like healthier options
- 1oz grain equivalent:
  - 1oz bread/bun
  - 1/2c cooked pasta, rice, cereal
  - 1oz dry cereal

Milk:

- Variety of fat contents allowed
- No flavor specifications

**ESHA Notes:**

- Ranchero beans = used baked beans

**HEI Notes:**

- Canned fruit/ tropical fruit does not count as whole fruit

Supplementary Table 2. Six Weeks of Portioned Lunch Menu for Grades K-5 Applying HHFKA Nutrition Standards

Week #: 1

| **Component** | **Mon (9/24/18)** | **Tues (9/25/18)** | **Wed (9/26/18 )** | **Thurs (9/27/18 )** | **Fri**  **(9/28/18 )** |
| --- | --- | --- | --- | --- | --- |
| **Fruit (1/2c)** | 1/2c apple slices | 1/2c canned peaches | 1/2c canned pineapple | 1/2c banana slices | 1/2c tropical fruit mix |
| **Veg (3/4c)** | 3/4c French fries | 3/4c ranchero beans | 1 med. baked potato, 1/2c cucumber, 1T ranch | 3/4c fresh carrots, 2T ranch | 1c romaine lettuce, 1T Italian dressing |
| **Meat (2oz or 14oz protein)** | 2oz ground beef | 2oz ground beef, 2T cheese sauce | 14oz protein between chili and cheese | 14oz protein from nuggets | 14oz protein from cheese pizza |
| **Grain (2oz on 4 days, 1oz on 1 day)** | 1 hamburger bun (WG) | 2oz nacho chips (WG) | 1oz chocolate chip cookie | 30g carbs between nuggets and roll, 1t butter | 30g carbs from cheese pizza |
| **Milk (1c)** | 1c low fat | 1c chocolate milk non fat | 1c low fat | 1c chocolate milk non fat | 1c low fat |

DG (1/2c): 1/2c RO (3/4c): 3/4c Legumes(1/2c): 3/4c Starchy(1/2c): 1.5c Other(1/2c): 1/2c

Week #: 2

| **Component** | **Mon (10/1/18)** | **Tues (10/2/18)** | **Wed (10/3/18)** | **Thurs (10/4/18)** | **Fri**  **(10/5/18)** |
| --- | --- | --- | --- | --- | --- |
| **Fruit (1/2c)** | 1/2c apple slices | 1/2c canned peaches | 1/2c canned pineapple | 1/2c banana slices | 1/2c tropical fruit mix |
| **Veg (3/4c)** | 3/4 c baked beans | 3/4c fresh celery  2T LF ranch | 1c tomato soup | 3/4c cheesy potatoes | 1c romaine lettuce, 1T Caesar dressing |
| **Meat (2oz or 14oz protein)** | 2oz ground beef | 14g protein between chili and cheddar cheese | 2oz American cheese | 14g protein chicken tender | 14oz protein from Pepperoni pizza |
| **Grain (2oz on 4 days, 1oz on 1 day)** | 1 hamburger bun (WG) | 2oz Fritos chips (WG) | 2oz bread (WG) | 30g CHO between chicken and waffles and 1T syrup | 30g CHO from cheese pizza |
| **Milk (1c)** | 1c low fat | 1c chocolate milk non fat | 1c low fat | 1c chocolate milk non fat | 1c low fat |

DG (1/2c): 3/4c RO (3/4c): 3/4c Legumes(1/2c): 3/4c Starchy(1/2c): 3/4c Other(1/2c): 3/4c

Week #: 3

| **Component** | **Mon (10/8/18)** | **Tues (10/9/18)** | **Wed (9/19/18)** | **Thurs (10/11/18)** | **Fri (10/12/18)** |
| --- | --- | --- | --- | --- | --- |
| **Fruit (1/2c)** | 1/2c apple slices | 1/2c canned peaches | 1/2c canned pineapple | 1/2c banana slices | 1/2c tropical fruit mix |
| **Veg (3/4c)** | 3/4c potato wedges | 3/4c refried beans | Caesar salad 1 c romaine, 1T Caesar dressing  1/2c marinara sauce | 3/4c fresh carrots, 2T ranch | 1c iceberg lettuce, 1T Italian dressing  2T pizza sauce |
| **Meat (2oz or 14oz protein)** | 2oz ground beef | 2oz ground beef (with 1/8 tsp taco seasoning) | 2oz ground beef | 14g protein chicken tender | 14g protein from moz cheese |
| **Grain (2oz on 4 days, 1oz on 1 day)** | 1 hamburger bun (WG) | 2oz tortillas (WG) | 1c spaghetti | 15g CHO between chicken and garlic toast (as needed) | 2oz flatbread |
| **Milk (1c)** | 1c low fat | 1c chocolate milk non fat | 1c low fat | 1c chocolate milk non fat | 1c low fat |

DG (1/2c): 1/2c RO (3/4c): 1 &1/8c Legumes(1/2c): 3/4c Starchy(1/2c): 3/4c Other(1/2c): 1/2c

Week #: 4

| **Component** | **Mon (10/15/18)** | **Tues (10/16/18)** | **Wed (11/7/18)** | **Thurs (11/8/18)** | **Fri**  **(11/9/18)** |
| --- | --- | --- | --- | --- | --- |
| **Fruit (1/2c)** | 1/2c apple slices | 1/2c canned peaches | 1/2c canned pineapple | 1/2c berries | 1/2c tropical fruit mix |
| **Veg (3/4c)** | 3/4c French fries | 3/4c ranchero beans | 3/4c broccoli, 2T ranch | 3/4c green beans, 1tsp butter | 3/4c sweet potato fries |
| **Meat (2oz or 14oz protein)** | 14g protein BBQ pork rib | 1.5oz chicken  3-4g prot protein black beans | 14g prot tangerine chicken | 4oz vanilla yogurt, 1oz mozzarella cheese stick | 14oz protein between refried beans and cheese |
| **Grain (2oz on 4 days, 1oz on 1 day)** | 2oz hoagie bun | 1.5oz tortilla WG, and 1/4c brown rice | Carbs from chicken + minimum 1/4c cooked rice | 1/4c Granola WG | 1.5oz tortilla WG, 1/4 c brown rice |
| **Milk (1c)** | 1c low fat | 1c chocolate milk non fat | 1c low fat | 1c chocolate milk non fat | 1c low fat |

DG (1/2c)__3/4___ RO (3/4c)__3/4___ Legumes(1/2c)___3/4__ Starchy(1/2c)__3/4___ Other(1/2c)__3/4___

Week #: 5

| **Component** | **Mon (11/12/18)** | **Tues (11/13/18)** | **Wed (11/14/18)** | **Thurs (11/15/18)** | **Fri (11/16/18)** |
| --- | --- | --- | --- | --- | --- |
| **Fruit (1/2c)** | 1/2c apple slices | 1/2c canned peaches | 1/2c canned pineapple | 1/2c banana slices | 1/2c tropical fruit mix |
| **Veg (3/4c)** | 3/4c cowboy beans | 3/4c corn, 1tsp butter | 1c lettuce, 1T LF ranch | 3/4c carrots, 2T LF ranch | 1c romaine, 1T Caesar dressing |
| **Meat (2oz or 14oz protein)** | 14g prot bread chicken | 14g protein from cheese pizza | 2oz ground beef, 1/8c sloppy Joe sauce | 2oz Swedish meatballs | 14oz protein from cheese |
| **Grain (2oz on 4 days, 1oz on 1 day)** | 2oz WG hamburger bun | 30g CHO from pizza | 2oz WG hamburger bun | 1/2c cooked rice | 30g carbs macaroni pasta |
| **Milk (1c)** | 1c low fat | 1c chocolate milk non fat | 1c low fat | 1c chocolate milk non fat | 1c low fat |

DG (1/2c)__3/4___ RO (3/4c)___3/4__ Legumes(1/2c)__3/4___ Starchy(1/2c)__3/4___ Other(1/2c)__3/4___

Week #: 6

| **Component** | **Mon (10/8/18)** | **Tues (10/9/18)** | **Wed (10/10/18)** | **Thurs (10/11/18)** | **Fri (10/12/18)** |
| --- | --- | --- | --- | --- | --- |
| **Fruit (1/2c)** | 1/2c apple slices | 1/2c canned peaches | 1/2c canned pineapple | 1/2c banana slices | 1/2c tropical fruit mix |
| **Veg (3/4c)** | 3/4c French fries | 3/4c refried beans | 3/4c steamed broccoli, 2 T cheese sauce | 1/4c carrots  1T LF ranch, 1/2c marinara | 1c lettuce, 1T Italian dressing |
| **Meat (2oz or 14oz protein)** | 2oz grilled chicken | 1oz shredded chicken, 1oz cheddar cheese | 14g prot grilled chicken leg | 2oz mozzarella cheese | 1.5oz shredded chicken, .5oz cheddar, 2T BBQ sauce |
| **Grain (2oz on 4 days, 1oz on 1 day)** | 2oz WG hamburger bun | 2oz WG tortilla | 1/2c cooked rice | 1c ziti | 2oz nacho chips (WG) |
| **Milk (1c)** | 1c low fat | 1c chocolate milk non fat | 1c low fat | 1c chocolate milk non fat | 1c low fat |

DG (1/2c)__3/4___ RO (3/4c)__3/4___ Legumes(1/2c)__3/4___ Starchy(1/2c)___3/4__ Other(1/2c)__3/4___

**Portioning Notes:**

- Week 3: Wednesday was substituted to match pattern

Fruits:

- Added a fruit pattern that was the same for each week (because the original menu did not have a fruit menu).
  - Picked variety of typical pattern of canned and fresh fruit.

Grains:

- Followed weekly pattern:
  - Two days of whole grain for one week
  - Three days of whole grain for following week
  - Alternate
- 1oz grain equivalent
  - 1oz bread/bun
  - 1/2c cooked pasta, rice, cereal
  - 1oz dry cereal
  - 1/4c granola
- If combination food (ex: pizza) there should be minimum of 30grams of carbohydrate to equal 2oz of grain.
- Plain roll served with 1tsp butter

Milk:

- Schoolchildren will typically pick chocolate milk
  - Served two out of five days per week to be conservative

Meats:

- Burger
  - Must be 80% lean/20% fat
- Cheese
  - Must be real cheese
  - Ex: cheese sauce does not count
- Hotdogs
  - Must be 80% muscle meat
- Lunch meats
  - Must be muscle meat
- If combination food: there should be 14grams of protein to equal 2oz of meat.

Vegetables:

- Depends on which standards need to be met.
  - Chose options that complement the entrée.
  - Sweet potato count as a starchy vegetable or Red-Orange food group
  - Legumes can be starchy or legume
- If fresh vegetables or salad:
  - Will be served with dressing:
    - 1Tbsp of appropriate dressing or dip 2Tbsp low-fat ranch
- Steamed or plain vegetables:
  - Add 1tsp of butter

Specific Food Items:

- Baked potato with toppings
  - 3/4c baked potato, 14oz protein between chili and cheese
- Burger will be most plain version
- Chicken burrito
  - 1.5oz chicken, 3-4g protein black beans, 1/8c rice, and 1.5oz tortilla
- Cowboy beans are equivalent to baked beans
- Nachos grande
  - 2oz ground beef, 2T cheese sauce, and 2oz nacho chips
- Pizza will be most plain version
- Ranchero beans are equivalent to baked beans
- Tacos will be most plain version
  - Beef and cheese
- Sloppy Joe
  - 2oz ground beef and 1/8c sloppy Joe sauce
- Spaghetti
  - 1/4c spaghetti served with 1/2c sauce
- Yogurt box
  - 4oz yogurt, 1oz string cheese, and 1/2c granola (2oz grain)

**Other notes:**

- Week 6 would have been the 3^rd^ week in the cycle
  - Reusing week 3 with cycle option to mimic option

Supplementary Table 3. Six Weeks of Portioned Lunch Menu for Grades K-5 Applying CNP Flexibilities Nutrition Standards

Week #: 1

| **Component** | **Mon (9/24/18)** | **Tues (9/25/18)** | **Wed (9/26/18 )** | **Thurs (9/27/18 )** | **Fri**  **(9/28/18 )** |
| --- | --- | --- | --- | --- | --- |
| **Fruit (1/2c)** | 1/2c apple slices | 1/2c canned peaches | 1/2c canned pineapple | 1/2c banana slices | 1/2c tropical fruit mix |
| **Veg (3/4c)** | 3/4c French fries | 3/4c ranchero beans | 1 med. baked potato, 1/2c cucumber, 1T ranch | 3/4c fresh carrots, 2T ranch | 1c romaine lettuce, 1T Italian dressing |
| **Meat (2oz or 14oz protein)** | 2oz ground beef | 2oz ground beef, 2T cheese sauce | 14oz protein between chili and cheese | 14oz protein from nuggets | 14oz protein from cheese pizza |
| **Grain (2oz on 4 days, 1oz on 1 day)** | 1 hamburger bun | 2oz nacho chips (WG) | 1oz chocolate chip cookie | 30g carbs between nuggets and roll, 1t butter | 30g carbs from cheese pizza |
| **Milk (1c)** | 1c low fat | 1c chocolate milk low fat | 1c low fat | 1c chocolate milk low fat | 1c low fat |

DG (1/2c): 1/2c RO (3/4c): 3/4c Legumes(1/2c): 3/4c Starchy(1/2c): 1.5c Other(1/2c): 1/2c

Week #: 2

| **Component** | **Mon (10/1/18)** | **Tues (10/2/18)** | **Wed (10/3/18)** | **Thurs (10/4/18)** | **Fri**  **(10/5/18)** |
| --- | --- | --- | --- | --- | --- |
| **Fruit (1/2c)** | 1/2c apple slices | 1/2c canned peaches | 1/2c canned pineapple | 1/2c banana slices | 1/2c tropical fruit mix |
| **Veg (3/4c)** | 3/4 c baked beans | 3/4c fresh celery  2 T LF ranch | 1c tomato soup | 3/4c cheesy potatoes | 1c romaine lettuce, 1T Caesar dressing |
| **Meat (2oz or 14oz protein)** | 2oz ground beef | 14g protein between chili and cheddar cheese | 2oz American cheese | 14g protein chicken tender | 14oz protein from Pepperoni pizza |
| **Grain (2oz on 4 days, 1oz on 1 day)** | 1 hamburger bun | 2oz Fritos chips (WG) | 2oz bread (WG) 1t butter | 30g CHO between chicken and waffles and 1T syrup | 30g CHO from cheese pizza |
| **Milk (1c)** | 1c low fat | 1c chocolate milk low fat | 1c low fat | 1c chocolate milk low fat | 1c low fat |

DG (1/2c): 3/4c RO (3/4c): 3/4c Legumes(1/2c): 3/4c Starchy(1/2c): 3/4c Other(1/2c): 3/4c

Week #: 3

| **Component** | **Mon (10/8/18)** | **Tues (10/9/18)** | **Wed (9/19/18)** | **Thurs (10/11/18)** | **Fri (10/12/18)** |
| --- | --- | --- | --- | --- | --- |
| **Fruit (1/2c)** | 1/2c apple slices | 1/2c canned peaches | 1/2c canned pineapple | 1/2c banana slices | 1/2c tropical fruit mix |
| **Veg (3/4c)** | 3/4c potato wedges | 3/4c refried beans | Caesar salad 1 c romaine, 1T Caesar dressing  1/2c marinara sauce | 3/4c fresh carrots, 2T ranch | 1c iceberg lettuce, 1T Italian dressing  2T pizza sauce |
| **Meat (2oz or 14oz protein)** | 2oz ground beef | 2oz ground beef (with 1/8 tsp taco seasoning) | 2oz ground beef | 14g protein chicken tender | 14g protein from moz cheese |
| **Grain (2oz on 4 days, 1oz on 1 day)** | 1 hamburger bun | 2oz tortillas (WG) | 1c spaghetti | 15g CHO between chicken and garlic toast (as needed) | 2oz flatbread |
| **Milk (1c)** | 1c low fat | 1c chocolate milk low fat | 1c low fat | 1c chocolate milk low fat | 1c low fat |

DG (1/2c): 1/2c RO (3/4c): 1 &1/8c Legumes(1/2c): 3/4c Starchy(1/2c): 3/4c Other(1/2c): 1/2c

Week #: 4

| **Component** | **Mon (10/15/18)** | **Tues (10/16/18)** | **Wed (11/7/18)** | **Thurs (11/8/18)** | **Fri**  **(11/9/18)** |
| --- | --- | --- | --- | --- | --- |
| **Fruit (1/2c)** | 1/2c apple slices | 1/2c canned peaches | 1/2c canned pineapple | 1/2c berries | 1/2c tropical fruit mix |
| **Veg (3/4c)** | 3/4c French fries | 3/4c ranchero beans | 3/4c broccoli, 2T ranch | 3/4c green beans, 1tsp butter | 3/4c sweet potato fries |
| **Meat (2oz or 14oz protein)** | 14g protein BBQ pork rib | 1.5oz chicken  3-4g prot protein black beans | 14g prot tangerine chicken | 4oz vanilla yogurt, 1oz mozzarella cheese stick | 14oz protein between refried beans and cheese |
| **Grain (2oz on 4 days, 1oz on 1 day)** | 2oz hoagie bun | 1.5oz tortilla WG, and 1/4c brown rice | Carbs from chicken + minim1um 1/4c cooked rice | 1/4c Granola | 1.5oz tortilla WG, 1/4 c brown rice |
| **Milk (1c)** | 1c low fat | 1c chocolate milk low fat | 1c low fat | 1c chocolate milk low fat | 1c low fat |

DG (1/2c)__3/4___ RO (3/4c)__3/4___ Legumes(1/2c)___3/4__ Starchy(1/2c)__3/4___ Other(1/2c)__3/4___

Week #: 5

| **Component** | **Mon (11/12/18)** | **Tues (11/13/18)** | **Wed (11/14/18)** | **Thurs (11/15/18)** | **Fri (11/16/18)** |
| --- | --- | --- | --- | --- | --- |
| **Fruit (1/2c)** | 1/2c apple slices | 1/2c canned peaches | 1/2c canned pineapple | 1/2c banana slices | 1/2c tropical fruit mix |
| **Veg (3/4c)** | 3/4c cowboy beans | 3/4c corn, 1tsp butter | 1c lettuce, 1T LF ranch | 3/4c carrots, 2T LF ranch | 1c romaine, 1T Caesar dressing |
| **Meat (2oz or 14oz protein)** | 14g prot bread chicken | 14g protein from cheese pizza | 2oz ground beef, 1/8c sloppy Joe sauce | 2oz Swedish meatballs | 14oz protein from cheese |
| **Grain (2oz on 4 days, 1oz on 1 day)** | 2oz hamburger bun | 30g CHO from pizza | 2oz WG hamburger bun | 1/2c cooked rice | 30g carbs macaroni pasta |
| **Milk (1c)** | 1c low fat | 1c chocolate milk low fat | 1c low fat | 1c chocolate milk low fat | 1c low fat |

DG (1/2c)__3/4___ RO (3/4c)___3/4__ Legumes(1/2c)__3/4___ Starchy(1/2c)__3/4___ Other(1/2c)__3/4___

Week #: 6

| **Component** | **Mon (10/8/18)** | **Tues (10/9/18)** | **Wed (10/10/18)** | **Thurs (10/11/18)** | **Fri (10/12/18)** |
| --- | --- | --- | --- | --- | --- |
| **Fruit (1/2c)** | 1/2c apple slices | 1/2c canned peaches | 1/2c canned pineapple | 1/2c banana slices | 1/2c tropical fruit mix |
| **Veg (3/4c)** | 3/4c French fries | 3/4c refried beans | 3/4c steamed broccoli, 2 T cheese sauce | 1/4c carrots  1T LF ranch, 1/2c marinara | 1c lettuce, 1T Italian dressing |
| **Meat (2oz or 14oz protein)** | 2oz grilled chicken | 1oz shredded chicken, 1oz cheddar cheese | 14g prot grilled chicken leg | 2oz mozzarella cheese | 1.5oz shredded chicken, .5oz cheddar, 2T BBQ sauce |
| **Grain (2oz on 4 days, 1oz on 1 day)** | 2oz hamburger bun | 2oz WG tortilla | 1/2c cooked rice | 1c ziti | 2oz nacho chips (WG) |
| **Milk (1c)** | 1c low fat | 1c chocolate milk low fat | 1c low fat | 1c chocolate milk low fat | 1c low fat |

DG (1/2c)__3/4___ RO (3/4c)__3/4___ Legumes(1/2c)__3/4___ Starchy(1/2c)___3/4__ Other(1/2c)__3/4___

**Portioning Notes:**

- Week 3: Wednesday was substituted to match pattern

Fruits:

- Added a fruit pattern that was the same for each week (because the original menu did not have a fruit menu).
  - Picked variety of typical pattern of canned and fresh fruit.

Grains:

- Followed weekly pattern:
  - Two days of whole grain for one week
  - Three days of whole grain for following week
  - Alternate
- 1oz grain equivalent
  - 1oz bread/bun
  - 1/2c cooked pasta, rice, cereal
  - 1oz dry cereal
  - 1/4c granola
- If combination food (ex: pizza) there should be minimum of 30grams of carbohydrate to equal 2oz of grain.
- Plain roll served with 1tsp butter

Milk:

- Schoolchildren will typically pick chocolate milk
  - Served two out of five days per week to be conservative

Meats:

- Burger
  - Must be 80% lean/20% fat
- Cheese
  - Must be real cheese
  - Ex: cheese sauce does not count
- Hotdogs
  - Must be 80% muscle meat
- Lunch meats
  - Must be muscle meat
- If combination food: there should be 14grams of protein to equal 2oz of meat.

Vegetables:

- Depends on which standards need to be met.
  - Chose options that complement the entrée.
  - Sweet potato count as a starchy vegetable or Red-Orange food group
  - Legumes can be starchy or legume
- If fresh vegetables or salad:
  - Will be served with dressing:
    - 1Tbsp of appropriate dressing or dip 2Tbsp low-fat ranch
- Steamed or plain vegetables:
  - Add 1tsp of butter

Specific Food Items:

- Baked potato with toppings
  - 3/4c baked potato, 14oz protein between chili and cheese
- Burger will be most plain version
- Chicken burrito
  - 1.5oz chicken, 3-4g protein black beans, 1/8c rice, and 1.5oz tortilla
- Cowboy beans are equivalent to baked beans
- Nachos grande
  - 2oz ground beef, 2T cheese sauce, and 2oz nacho chips
- Pizza will be most plain version
- Ranchero beans are equivalent to baked beans
- Tacos will be most plain version
  - Beef and cheese
- Sloppy Joe
  - 2oz ground beef and 1/8c sloppy Joe sauce
- Spaghetti
  - 1/4c spaghetti served with 1/2c sauce
- Yogurt box
  - 4oz yogurt, 1oz string cheese, and 1/2c granola (2oz grain)

**Other notes:**

- Week 6 would have been the 3^rd^ week in the cycle
  - Reusing week 3 with cycle option to mimic option

Supplementary Table 4. Six Weeks of Portioned Lunch Menu for Grades K-5 Applying BP Nutrition Standards

Week #: 1

| **Component** | **Mon (9/24/18)** | **Tues (9/25/18)** | **Wed (9/26/18)** | **Thurs (9/27/18)** | **Fri**  **(9/28/18)** |
| --- | --- | --- | --- | --- | --- |
| **Fruit (1/2c)** | 1/2c fresh apple | 1/2c fresh grapes | 1/2c banana | 1/2c fresh pineapple | 1/2c fresh pear |
| **Veg (3/4c)** | 3/4c homemade baked French fries | 1/4c lettuce, 1/4c tomato,  1/4c salsa | 1 med. baked potato, 1/2c cucumber, 1T Greek yogurt ranch | 3/4c fresh carrots, 2T Greek yogurt ranch | 1c romaine lettuce, 1T Italian dressing ,  1/8c pizza sauce |
| **Meat (2oz or 14oz protein)** | 2oz ground beef (93% lean) | 1oz LF cheddar cheese, 7g protein black beans | 14oz protein between turkey chili and LF cheddar cheese (1/2c chili + 1/2oz cheese) | 14oz protein from homemade WG nuggets | 2oz LF mozzarella cheese |
| **Grain (2oz on 4 days, 1oz on 1 day)** | 1 hamburger bun (WG) | 2oz baked unsalted Tostitos chips (WG) | 1oz cornbread (WG) | 30g carbs between nuggets and roll (WG) | 30g carbs WG from pizza crust |
| **Milk (1c)** | 1c 1% plain | 1c 1% plain | 1c 1% plain | 1c 1% plain | 1c 1% plain |

DG (1/2c)_____ RO (3/4c)_____ Legumes(1/2c)_____ Starchy(1/2c)_____ Other(1/2c)_____

Week #: 2

| **Component** | **Mon (10/1/18)** | **Tues (10/2/18)** | **Wed (10/3/18)** | **Thurs (10/4/18)** | **Fri**  **(10/5/18)** |
| --- | --- | --- | --- | --- | --- |
| **Fruit (1/2c)** | 1/2c fresh apple | 1/2c fresh grapes | 1/2c banana | 1/2c fresh pineapple | 1/2c fresh pear |
| **Veg (3/4c)** | 3/4 c dried reconstituted black beans | 3/4c fresh celery  2T LF Greek yogurt ranch | 1c low sodium tomato soup | 3/4c oven roasted potatoes | 1c romaine lettuce, 1T Italian dressing |
| **Meat (2oz or 14oz protein)** | 2oz ground beef (93% lean) | 14g protein between turkey chili and LF cheddar cheese (1/2c chili + ½ oz cheese) | 2oz LF cheddar, mozzarella, & Swiss cheese | 14g protein homemade chicken tender | 2oz LF mozzarella cheese |
| **Grain (2oz on 4 days, 1oz on 1 day)** | 1 hamburger bun (WG) | 2oz baked unsalted Tostitos chips (WG) | 2oz bread (WG) | 30g CHO between chicken and waffles (WG) and 1T light syrup | 30g CHO from cheese pizza (WG) |
| **Milk (1c)** | 1c 1% plain | 1c 1% plain | 1c 1% plain | 1c 1% plain | 1c 1% plain |

DG (1/2c)_____ RO (3/4c)_____ Legumes(1/2c)_____ Starchy(1/2c)_____ Other(1/2c)_____

Week #: 3

| **Component** | **Mon (10/8/18)** | **Tues (10/8/18)** | **Wed (9/19/18)** | **Thurs (10/11/18)** | **Fri  (10/12/18)** |
| --- | --- | --- | --- | --- | --- |
| **Fruit (1/2c)** | 1/2c fresh apple | 1/2c fresh grapes | 1/2c banana | 1/2c fresh pineapple | 1/2c fresh pear |
| **Veg (3/4c)** | 3/4c homemade baked potato wedges | 3/4c homemade refried beans | 1 c romaine, 1T Italian dressing  1/2c low sodium marinara sauce | 3/4c fresh carrots, 2T LF Greek yogurt ranch | 1c iceberg lettuce, 1T Italian dressing  2T low sodium pizza sauce |
| **Meat (2oz or 14oz protein)** | 2oz ground beef (93% lean) | 2oz shredded chicken (with 1/8 tsp taco seasoning) | 2oz ground turkey | 14g protein homemade chicken tender | 14g protein from mozzarella cheese |
| **Grain (2oz on 4 days, 1oz on 1 day)** | 1 hamburger bun (WG) | 2oz tortillas (WG) | 1c spaghetti (WG) | 15g CHO between chicken and WG roll (as needed) | 2oz flatbread (WG) |
| **Milk (1c)** | 1c 1% plain | 1c 1% plain | 1c 1% plain | 1c 1% plain | 1c 1% plain |

DG (1/2c)_____ RO (3/4c)_____ Legumes(1/2c)_____ Starchy(1/2c)_____ Other(1/2c)_____

Week #: 4

| **Component** | **Mon (10/15/18)** | **Tues (10/16/18)** | **Wed (11/7/18)** | **Thurs (11/8/18)** | **Fri**  **(11/9/18)** |
| --- | --- | --- | --- | --- | --- |
| **Fruit (1/2c)** | 1/2c fresh apple | 1/2c fresh grapes | 1/2c banana | 1/2c fresh pineapple | 1/2c fresh pear |
| **Veg (3/4c)** | 3/4c homemade baked French fries | 3/4c dried reconstituted pinto beans | 3/4c fresh broccoli, 2T Greek yogurt ranch | 3/4c frozen green beans | 3/4c homemade baked sweet potato fries |
| **Meat (2oz or 14oz protein)** | 2oz protein BBQ chicken, 1T low sodium BBQ sauce | 1.5oz chicken,  3-4g protein reconstituted black beans (1oz.) | 14g protein teriyaki grilled chicken, 1/2T low sodium teriyaki sauce | 4oz vanilla yogurt, 1oz LF mozzarella cheese stick | 14oz protein between dried reconstituted black beans and LF cheddar cheese |
| **Grain (2oz on 4 days, 1oz on 1 day)** | 2oz hoagie bun (WG) | 1.5oz tortilla WG, and 1/4c brown rice | 1c cooked brown rice | 1/4c Granola WG | 1.5oz tortilla WG, 1/4c cooked brown rice |
| **Milk (1c)** | 1c 1% plain | 1c 1% plain | 1c 1% plain | 1c 1% plain | 1c 1% plain |

DG (1/2c)_____ RO (3/4c)_____ Legumes(1/2c)_____ Starchy(1/2c)_____ Other(1/2c)_____

Week #: 5

| **Component** | **Mon (11/12/18)** | **Tues (11/13/18)** | **Wed (11/14/18)** | **Thurs (11/15/18)** | **Fri (11/16/18)** |
| --- | --- | --- | --- | --- | --- |
| **Fruit (1/2c)** | 1/2c fresh apple | 1/2c fresh grapes | 1/2c banana | 1/2c fresh pineapple | 1/2c fresh pear |
| **Veg (3/4c)** | 3/4c homemade cowboy beans (salad) | 3/4c frozen corn  2T low sodium pizza sauce | 1c lettuce, 1T LF Greek yogurt ranch | 3/4c carrots, 2T LF Greek yogurt ranch | 1c romaine, 1T Italian dressing |
| **Meat (2oz or 14oz protein)** | 2oz grilled chicken | 14g protein from LF mozzarella cheese | 2oz ground turkey, 1/8c sloppy joe sauce | 2oz Swedish meatball (93% lean) | 14oz protein from LF cheddar cheese |
| **Grain (2oz on 4 days, 1oz on 1 day)** | 2oz hamburger bun (WG) | 30g CHO from cheese pizza (WG) | 1 hamburger bun (WG) | 1/2c cooked brown rice | 1/2c WG macaroni pasta |
| **Milk (1c)** | 1c 1% plain | 1c 1% plain | 1c 1% plain | 1c 1% plain | 1c 1% plain |

DG (1/2c)_____ RO (3/4c)_____ Legumes(1/2c)_____ Starchy(1/2c)_____ Other(1/2c)_____

Week #: 6

| **Component** | **Mon**  **(10/8/18)** | **Tues (10/9/18)** | **Wed (10/10/18)** | **Thurs (10/11/18)** | **Fri (10/12/18)** |
| --- | --- | --- | --- | --- | --- |
| **Fruit (1/2c)** | 1/2c fresh apple | 1/2c fresh grapes | 1/2c banana | 1/2c fresh pineapple | 1/2c fresh pear |
| **Veg (3/4c)** | 3/4c homemade baked French fries | 3/4c homemade refried beans | 3/4c frozen, steamed broccoli | 1/4c carrots  1T LF ranch, 1/2c low sodium marinara sauce | 1c lettuce, 1T Italian dressing |
| **Meat (2oz or 14oz protein)** | 2oz grilled chicken | 1oz shredded chicken, 1oz LF cheddar cheese | 14g protein grilled chicken leg | 2oz LF mozzarella cheese | 1.5oz shredded chicken, 0.5oz LF cheddar cheese, 2T low Na BBQ sauce |
| **Grain (2oz on 4 days, 1oz on 1 day)** | 2oz hamburger bun (WG) | 2oz WG tortilla | 1/2c cooked brown rice | 1c WG ziti | 2oz nacho chips (WG) |
| **Milk (1c)** | 1c 1% plain | 1c 1% plain | 1c 1% plain | 1c 1% plain | 1c 1% plain |

DG (1/2c)_____ RO (3/4c)_____ Legumes(1/2c)_____ Starchy(1/2c)_____ Other(1/2c)_____

**Portioning Notes:**

- Week 3: Wednesday was substituted to match pattern

Fruits:

- Added a fruit pattern that was the same for each week (because the original menu did not have a fruit menu).
  - Picked variety of commonly used fresh fruit in school

Grains:

- Each item was whole grain
- 1oz grain equivalent
  - 1oz bread/bun
  - 1/2c cooked pasta, rice, cereal
  - 1oz dry cereal
  - 1/4c granola
- If combination food (ex: pizza) there should be minimum of 30grams of carbohydrate to equal 2oz of grain.

Meat:

- Lean options and cooking methods (>80% lean/20% fat beef)
- Substituted beef for leaner options if, more than once per week with appropriate item to match meal (increased variety of proteins used).
- Low-fat cheeses
- If combination food: there should be 14grams of protein to equal 2oz of meat.

Milk:

- No flavored milk
- All low-fat content

Vegetables:

- Depends on which standards need to be met.
  - Chose options that complement the entrée.
  - Sweet potato count as a starchy vegetable or Red-Orange food group
  - Legumes can be starchy or legume
- If fresh vegetables or salad:
  - Will be served with dressing:
    - 1Tbsp of clear appropriate dressing or dip 2Tbsp non-fat Greek yogurt ranch
- Steamed or plain vegetables:
  - Add 1tsp of butter

**ESHA Notes**

- Homemade baked French fries or oven roasted potatoes
  - 1 baked potato and 1/8 tsp. oil
- Greek yogurt ranch
  - Nonfat plain Greek yogurt, ranch herb recipe would be added, but provides no nutritional value, so only yogurt was added
- Whole grain chicken nuggets or tenders
  - 2oz grilled chicken, 1Tbsp egg beaters, 1Tbsp whole wheat bread crumbs
- Used healthy recipe for Swedish sauce and mac and cheese
